# Supplementary material for: Potent Antibacterial Prenylated Acetophenones from the Australian Endemic Plant Acronychia crassipetala
Source: Antibiotics (Basel). 2020 Aug 6;9(8):487. doi: 10.3390/antibiotics9080487 (PMC7460405; doi:10.3390/antibiotics9080487)
Supplement: Supplementary file 1 [file antibiotics-09-00487-s001.pdf]

Potent Anti-bacterial Prenylated Acetophenones from the Australian Endemic Plant *Acronychia crassipetala*

Trong D. Tran,[1] Malin A. Olsson,[2] David J. McMillan,[2] Jason K. Cullen,[3] Peter G. Parsons,[3] Paul W. Reddell,[4] and Steven M. Ogbourne[1,*]

[1] GeneCology Research Centre, School of Science and Engineering, University of the Sunshine Coast, Maroochydore DC, Queensland 4558, Australia.
[2] School of Health and Sports Sciences, University of the Sunshine Coast, Maroochydore DC, Queensland 4558, Australia.
[3] QIMR Berghofer Medical Research Institute, Locked Bag 2000, PO Royal Brisbane Hospital, Queensland 4029, Australia.
[4] QBiotics Limited, PO Box 1, Yungaburra, Queensland 4884, Australia.

Supplementary materials

Figure S1. ^{1}H Spectrum of **1** in DMSO-d_6
Figure S2. ^{13}C Spectrum of **1** in DMSO-d_6
Figure S3. HSQC Spectrum of **1** in DMSO-d_6
Figure S4. COSY Spectrum of **1** in DMSO-d_6
Figure S5. HMBC Spectrum of **1** in DMSO-d_6
Figure S6. NOESY Spectrum of **1** in DMSO-d_6
Figure S7. ^{1}H Spectrum of **2** in DMSO-d_6
Figure S8. ^{13}C Spectrum of **2** in DMSO-d_6
Figure S9. HSQC Spectrum of **2** in DMSO-d_6
Figure S10. COSY Spectrum of **2** in DMSO-d_6
Figure S11. HMBC Spectrum of **2** in DMSO-d_6
Figure S12. NOESY Spectrum of **2** in DMSO-d_6
Figure S13. HRMS spectrum of **1**
Figure S14. HRMS spectrum of **2**
Table S1. Antibacterial activity towards Gram-negative bacteria of **1**-**2**

Figure S1. ^{1}H Spectrum of **1** in DMSO-d_6

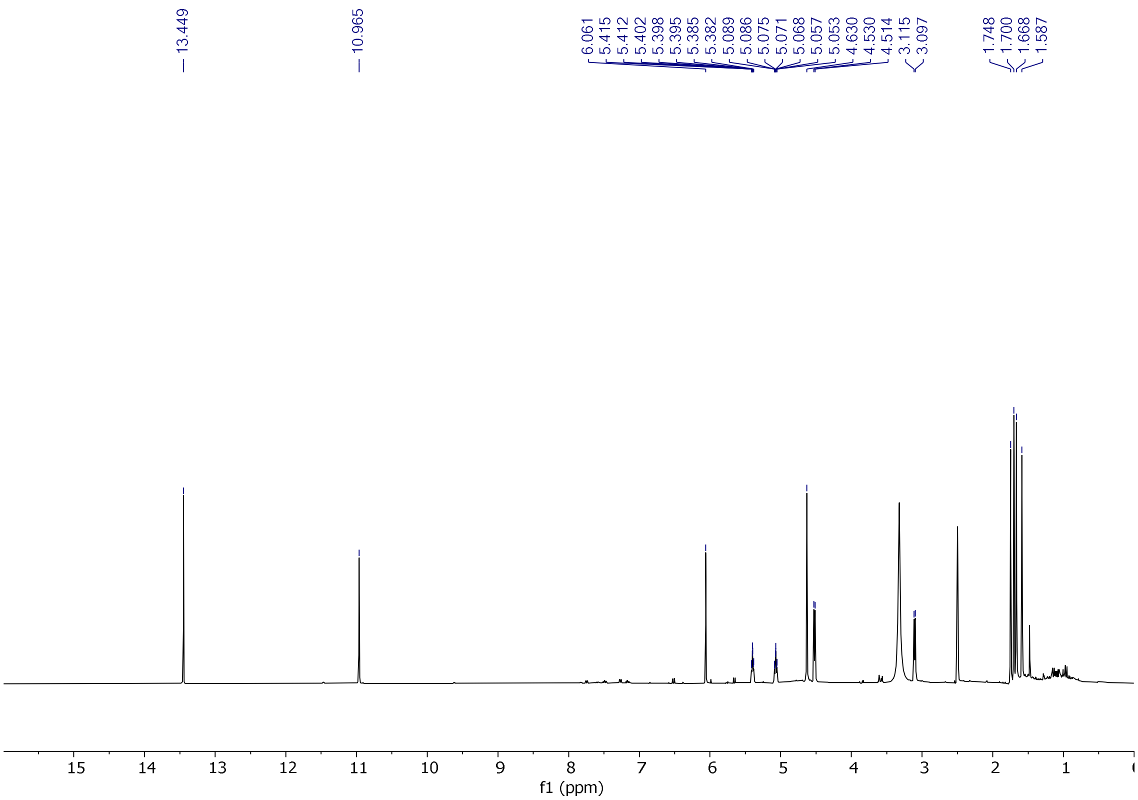

Figure S2. ^{13}C Spectrum of **1** in DMSO-d_6

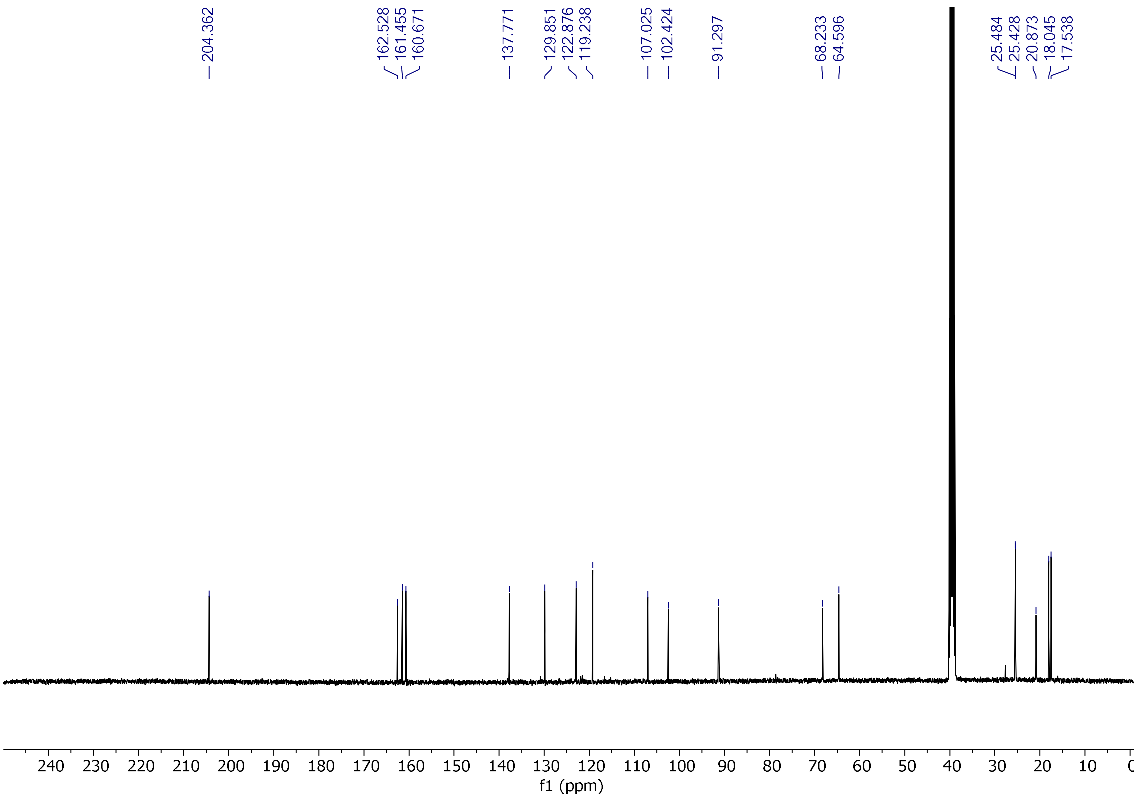

Figure S3. HSQC Spectrum of **1** in DMSO-d_6

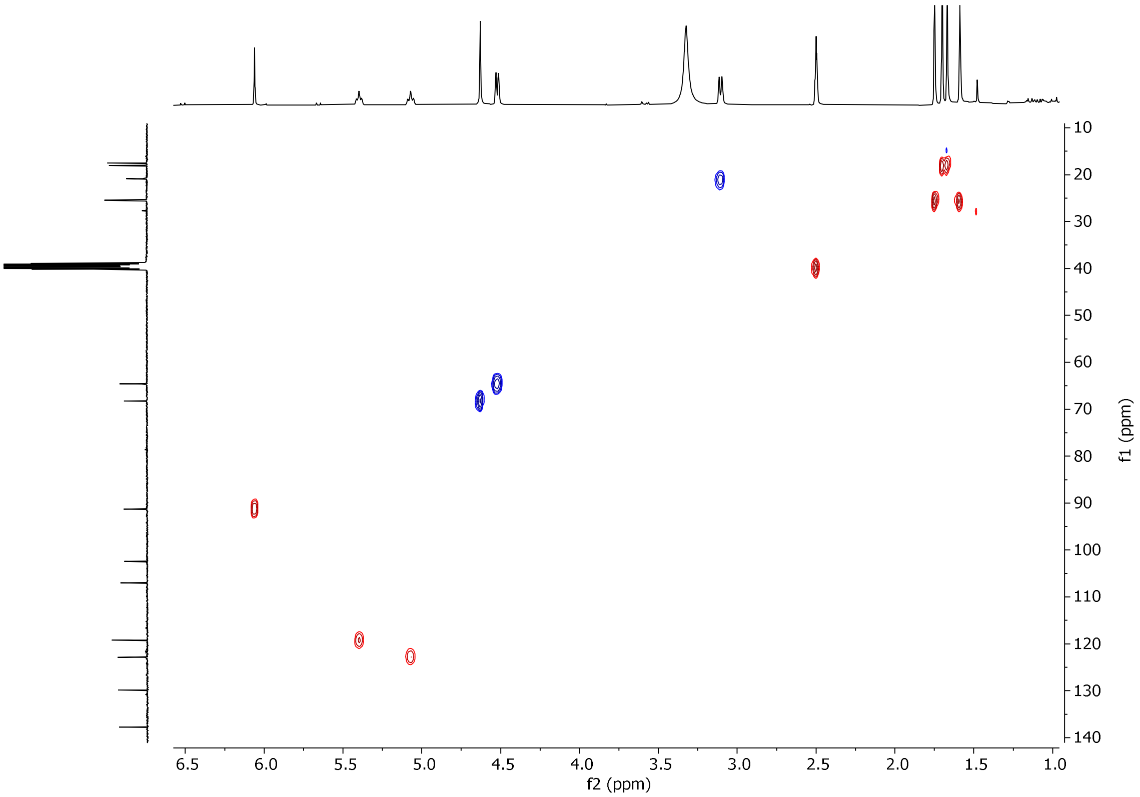

Figure S4. COSY Spectrum of **1** in DMSO-d_6

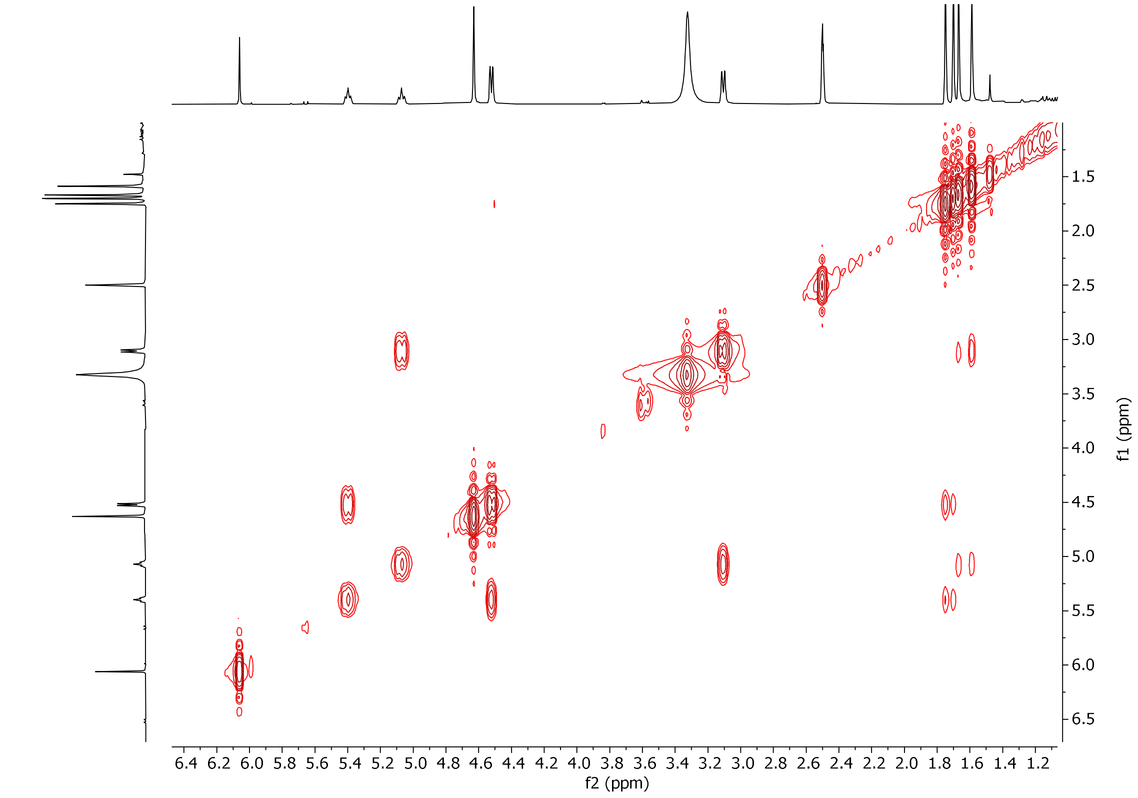

Figure S5. HMBC Spectrum of **1** in DMSO-d_6

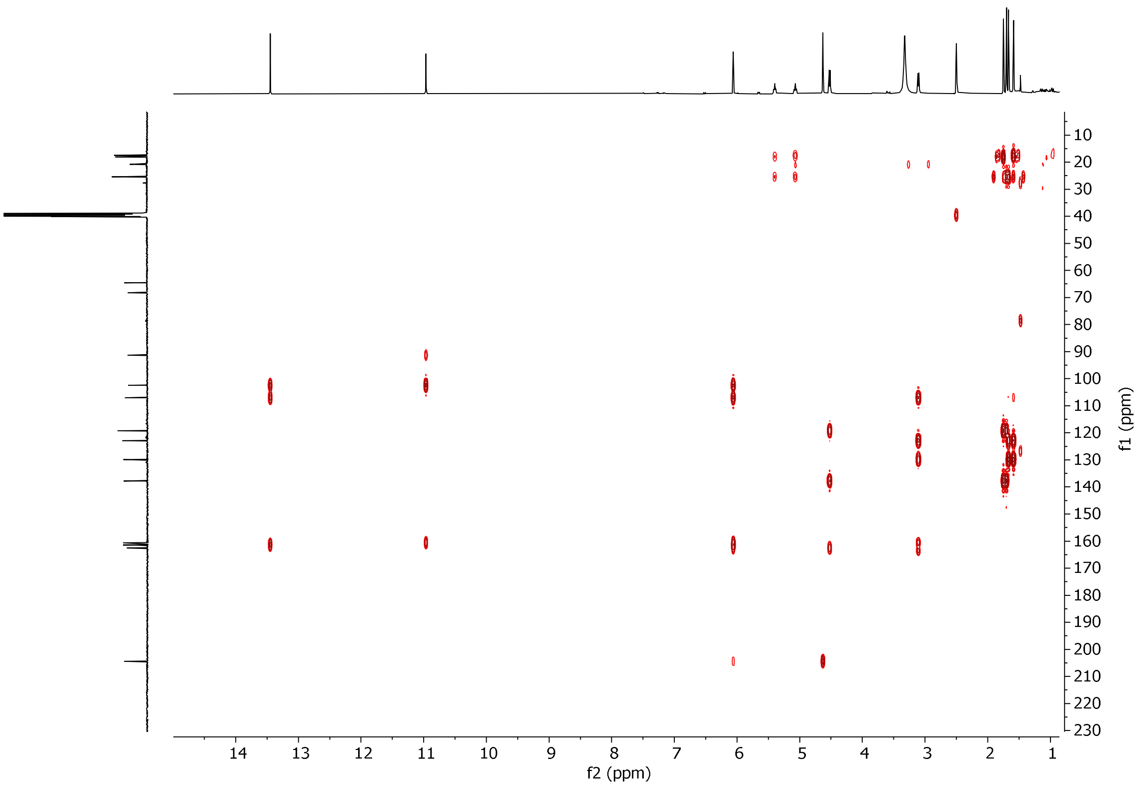

Figure S6. NOESY Spectrum of **1** in DMSO-d_6

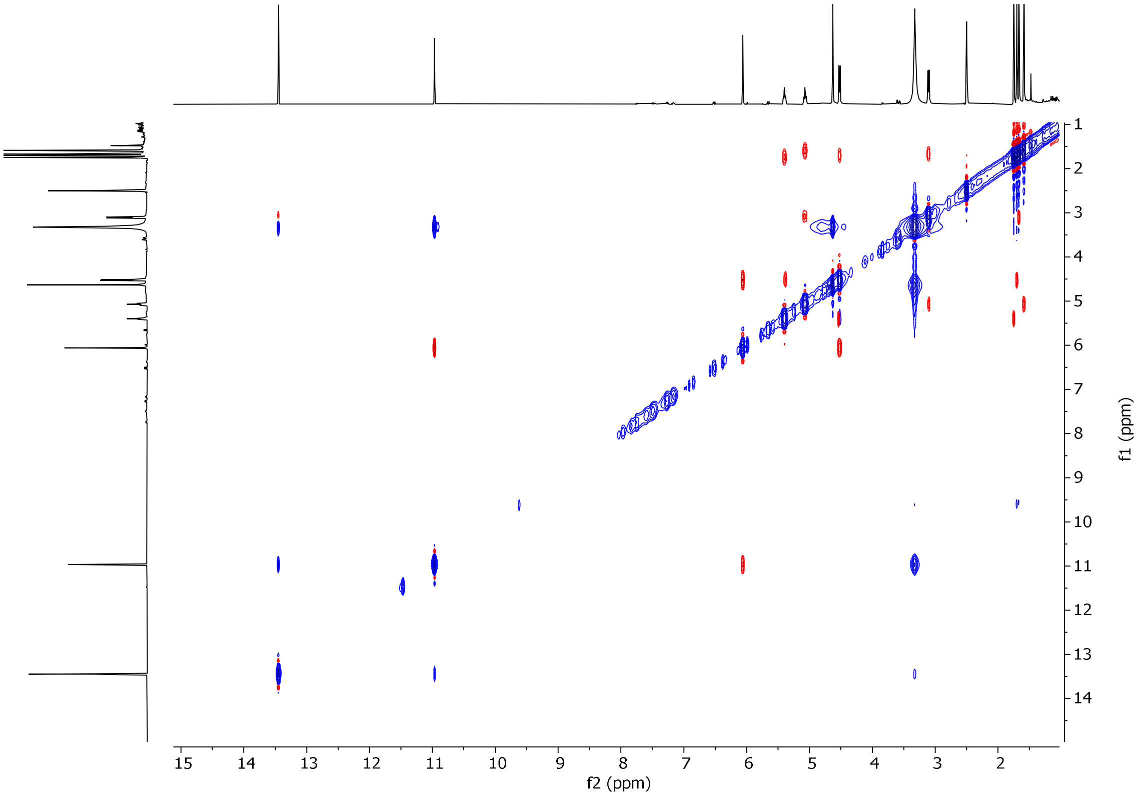

Figure S7. ^{1}H Spectrum of **2** in DMSO-d_6

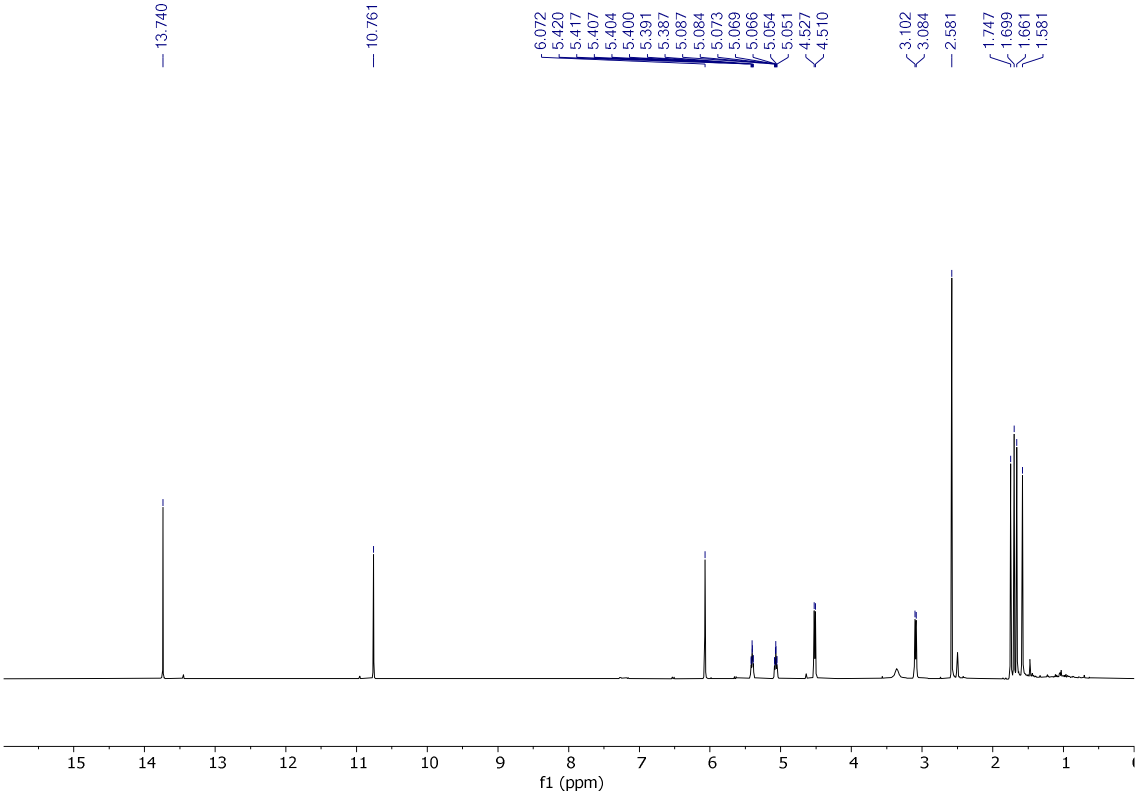

Figure S8. ^{13}C Spectrum of **2** in DMSO-d_6

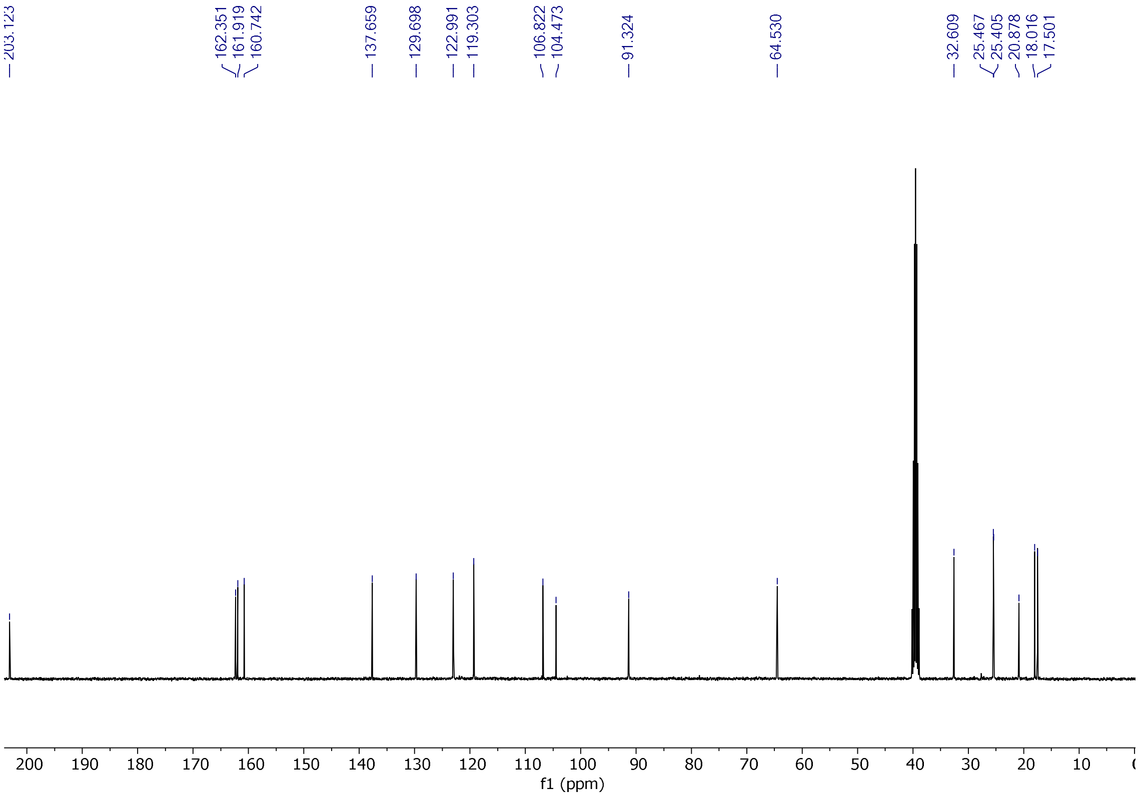

Figure S9. HSQC Spectrum of **2** in DMSO-d_6

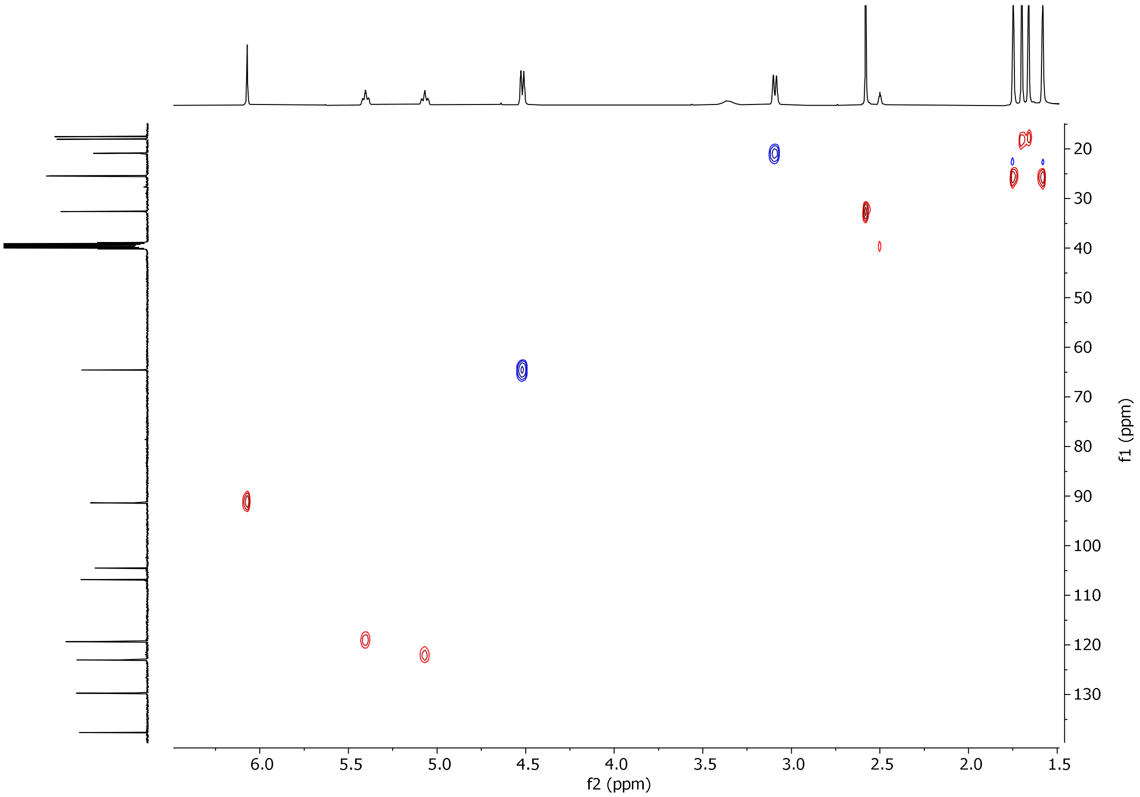

Figure S10. COSY Spectrum of **2** in DMSO-d_6

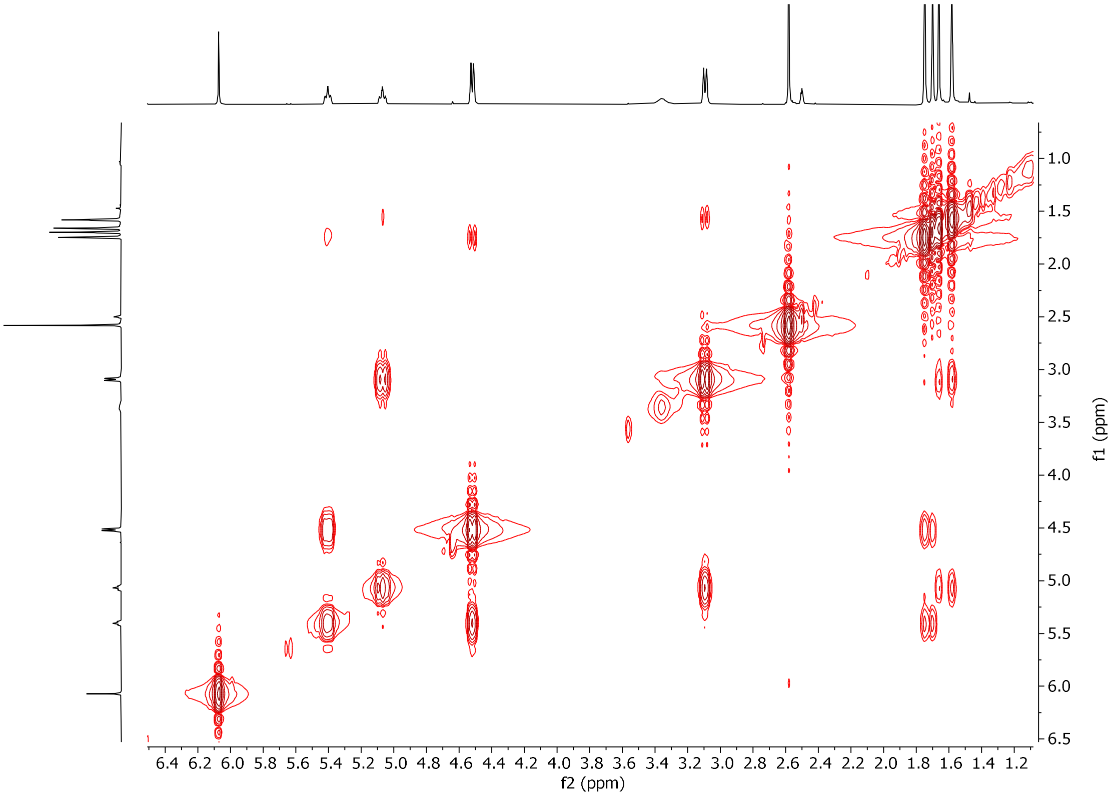

Figure S11. HMBC Spectrum of **2** in DMSO-d_6

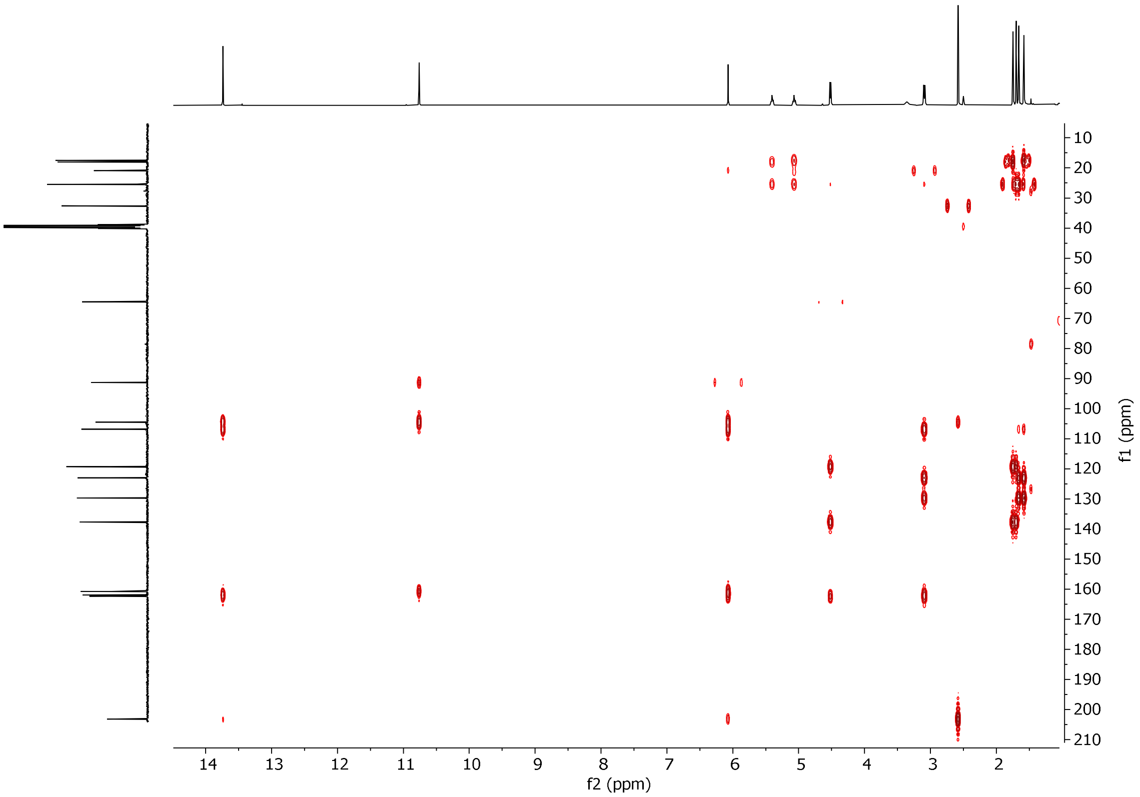

Figure S12. NOESY Spectrum of **2** in DMSO-d_6

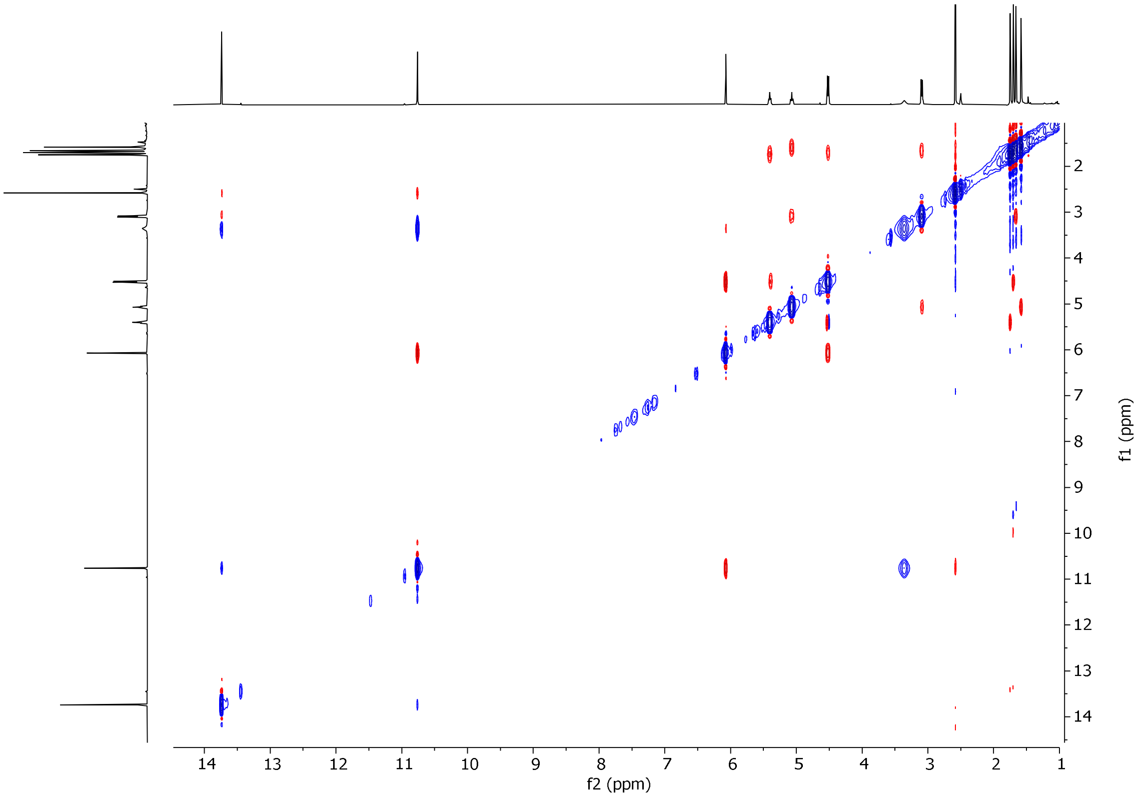

Figure S13. HRMS spectrum of **1**

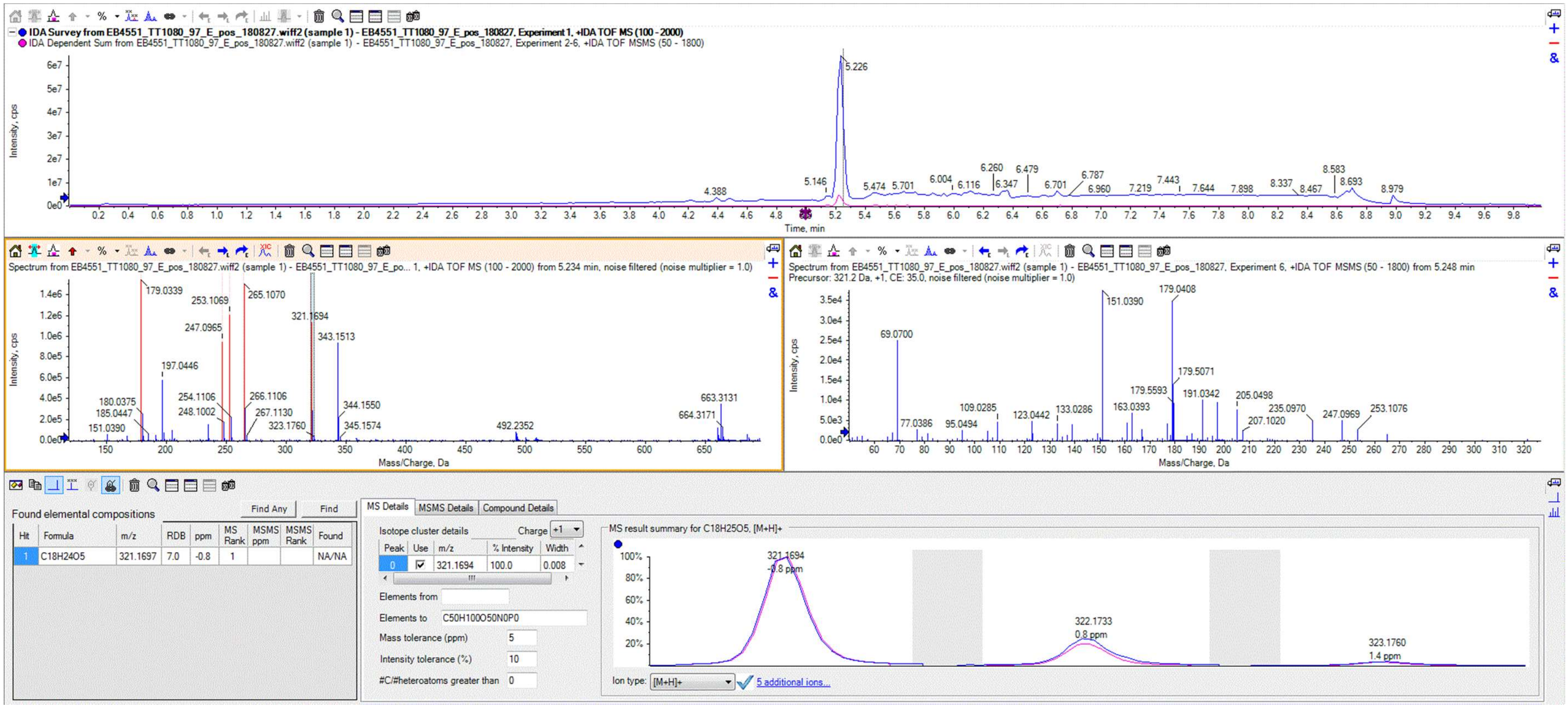

Figure S14. HRMS spectrum of **2**

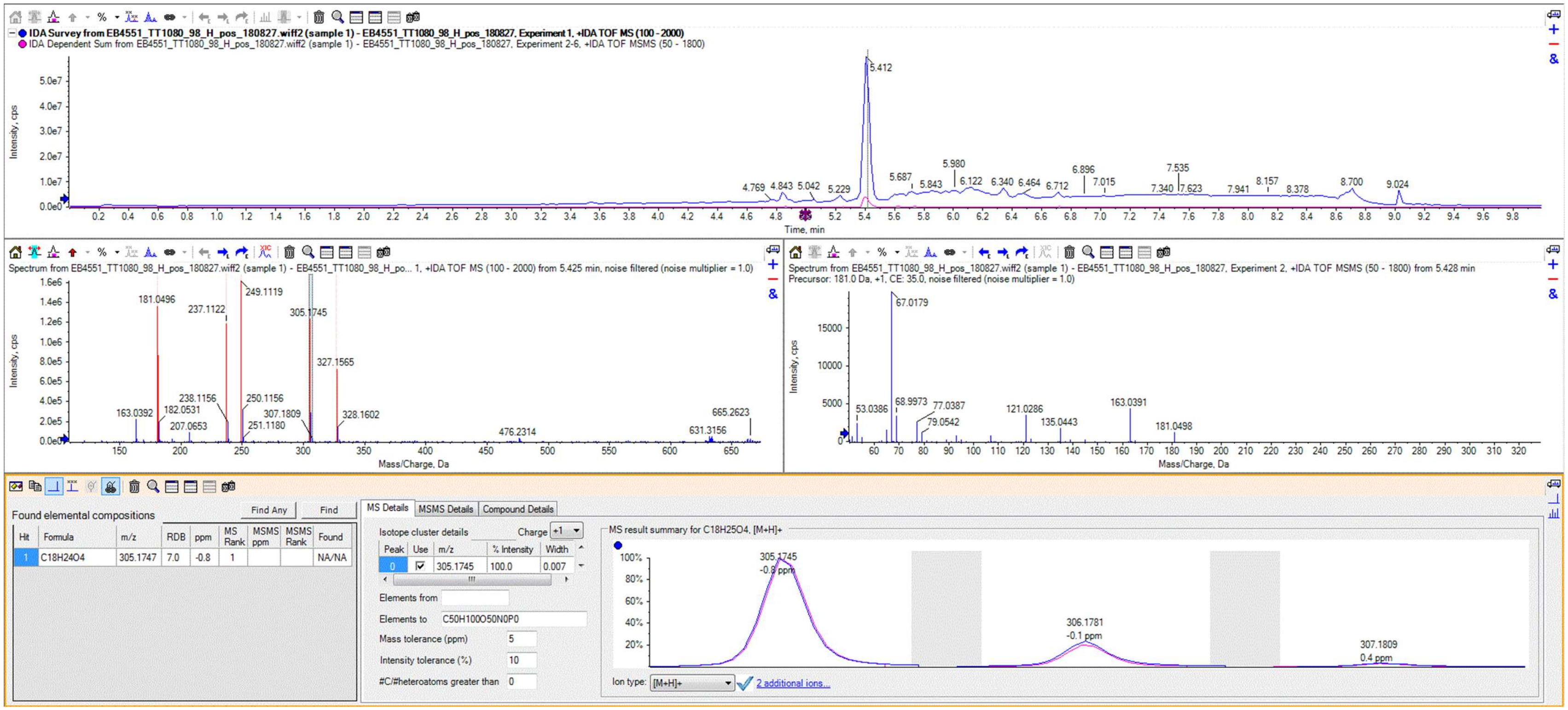

Table S1. Antibacterial activity towards Gram-negative bacteria of **1-2***

	MIC_{75} (µg/ml)							
	K. pneumoniae 13883	*K. pneumoniae* 12657	*A. baumannii* 19606	*A. baumannii* 17978	*P. aeruginosa* 10145	*P. aeruginosa* 49189	*E. aerogenes* 13048	*E. cloacae* 13047
**1**	*na*	*na*	*na*	*na*	*na*	*na*	*na*	*na*
**2**	*na*	*na*	*na*	*na*	*na*	*na*	*na*	*na*
**Chloramphenicol**	0.7813	1.5625	25	12.5	-	-	1.5625	1.5625
**Kanamycin**	-	-	-	-	12.5	25	-	-

* MIC_{75} (µg/ml) were tested in three independent experiments with triplicate determinations for each concentration.

na: Not active at the maximum tested concentration (50 µg/ml)
